# Calcium‐Based Antimicrobial Peptide Compounds as a Novel Treatment for Chronic Cheilitis: A Controlled Clinical Trial

**DOI:** 10.1111/jocd.71239

**Published:** 2026-11-08

**Authors:** Hyunsuk Ji, Tao Cai, Shuang Chen, Jianxia Xiong, Siyuan Geng, Aldi Dayu Saputra

**Affiliations:** ^1^ Department of Dermatology The First Affiliated Hospital of Chongqing Medical University Chongqing China

**Keywords:** calcium‐based antimicrobial peptides, chronic cheilitis, maintenance therapy, tacrolimus, tolerability

## Abstract

**Background:**

Chronic cheilitis refers to recurrent inflammation of the lips lasting for 3 months or longer. Commonly managed with topical corticosteroids or tacrolimus; however, these medications carry a risk of side effects. Calcium‐based antimicrobial peptide compounds (CAPCS) contain natural calcium derivatives and antimicrobial peptides that provide anti‐inflammatory and barrier‐supporting effects. We explored whether CAPCS lip balm could serve as an alternative to tacrolimus 0.03% for treating chronic cheilitis.

**Methods:**

A total of 52 patients with chronic cheilitis participated in this double‐blind, controlled trial and were allocated to use CAPCS lip balm (*n* = 26) or 0.03% tacrolimus ointment (*n* = 26) for a period of 4 weeks. Clinical assessments were performed at baseline, Week 2, and Week 4. A 3‐month follow‐up visit was conducted to evaluate recurrence or sustained improvement.

**Results:**

Forty‐five patients completed the 4‐week treatment. Both groups showed marked improvement from baseline by Week 4 (*p* < 0.001), although the tacrolimus group showed a faster reduction at Week 2 (4.4 ± 4.3 vs. 6.7 ± 4.0, *p* = 0.028). CAPCS showed good tolerability, with two cases of mild adverse events (7.7%), in contrast to six events (23.1%) observed in the tacrolimus group. At the 3‐month follow‐up, patients who continued CAPCS use showed sustained improvement, while recurrence was more common among those who stopped treatment.

**Conclusions:**

CAPCS lip balm demonstrated comparable efficacy to tacrolimus 0.03% ointment, was better tolerated, and may serve as a safe alternative or a maintenance option for chronic cheilitis.

## 1 Introduction

Chronic cheilitis is a recurrent inflammatory disease of the lips that affects not only their appearance but also daily activities such as eating, thereby reducing patients' overall quality of life. The cause of cheilitis is complex and involves multiple factors [1], and its various subtypes often exhibit overlapping clinical features, which has hindered the development of a consistent and universally agreed‐upon classification system [2]. Contributing factors may include sun exposure, contact with or ingestion of certain substances, infections, harmful habits, and nutritional deficiencies. Additionally, lip symptoms can occur in association with systemic diseases [1, 2].

Dermatologists and oral disease specialists primarily diagnose cheilitis [3]. Cheilitis is examined in multiple medical fields, but dermatologists focus more on pharmacological treatments. In particular, corticosteroids and tacrolimus are the two most commonly employed topical therapies [3]. Topical corticosteroids are used in the treatment of various skin conditions, and their efficacy is already widely recognized. However, prolonged use of medium‐ or high‐potency corticosteroids is not recommended due to the potential for various adverse effects [4, 5]. Topical tacrolimus, a calcineurin inhibitor, is used to treat moderate to severe atopic dermatitis, and numerous reports support its efficacy and safety [6]. Additionally, it is employed in the treatment of various forms of cheilitis, and the literature addressing its effectiveness is readily accessible [7, 8, 9]. Nevertheless, common side effects such as a burning sensation and itching cause discomfort for some patients, leading them to discontinue the medication. Thus, there is a need for alternative therapies that can avoid the adverse effects associated with current treatments.

Because the lip has a weak barrier and is prone to repeated inflammation and itching, treatments that support the barrier, reduce inflammation, and relieve itching could be helpful [10]. Calcium‐based antimicrobial peptide compounds (CAPCS) are made from natural calcium and antimicrobial peptides (AMPs) and have antibacterial, anti‐inflammatory, and antipruritic effects [11]. CAPCS have shown potential in mild to moderate atopic dermatitis [12], but little is known about its effectiveness and safety for cheilitis. Therefore, we conducted a controlled clinical trial to evaluate a lip balm containing CAPCS in patients with chronic cheilitis, compared with 0.03% tacrolimus ointment.

## 2 Methods

### 2.1 Study Design

This double‐blind, positive‐controlled study with randomized allocation enrolled 52 patients with chronic cheilitis (Figure 1). They were recruited from the dermatology outpatient clinic of a single center between February 2024 and April 2025. In accordance with the classification proposed by Lugović‐Mihić et al., only patients with chronic cases of mostly reversible cheilitis were included [2]. All participants had experienced recurrent lip inflammation for at least 3 months and exhibited various clinical signs and symptoms, including redness, dryness, scaling, erosion, or crusting; clinical manifestations of different subtypes sometimes overlapped or occurred simultaneously in a single patient. The individual characteristics varied from case to case.

**FIGURE 1:**
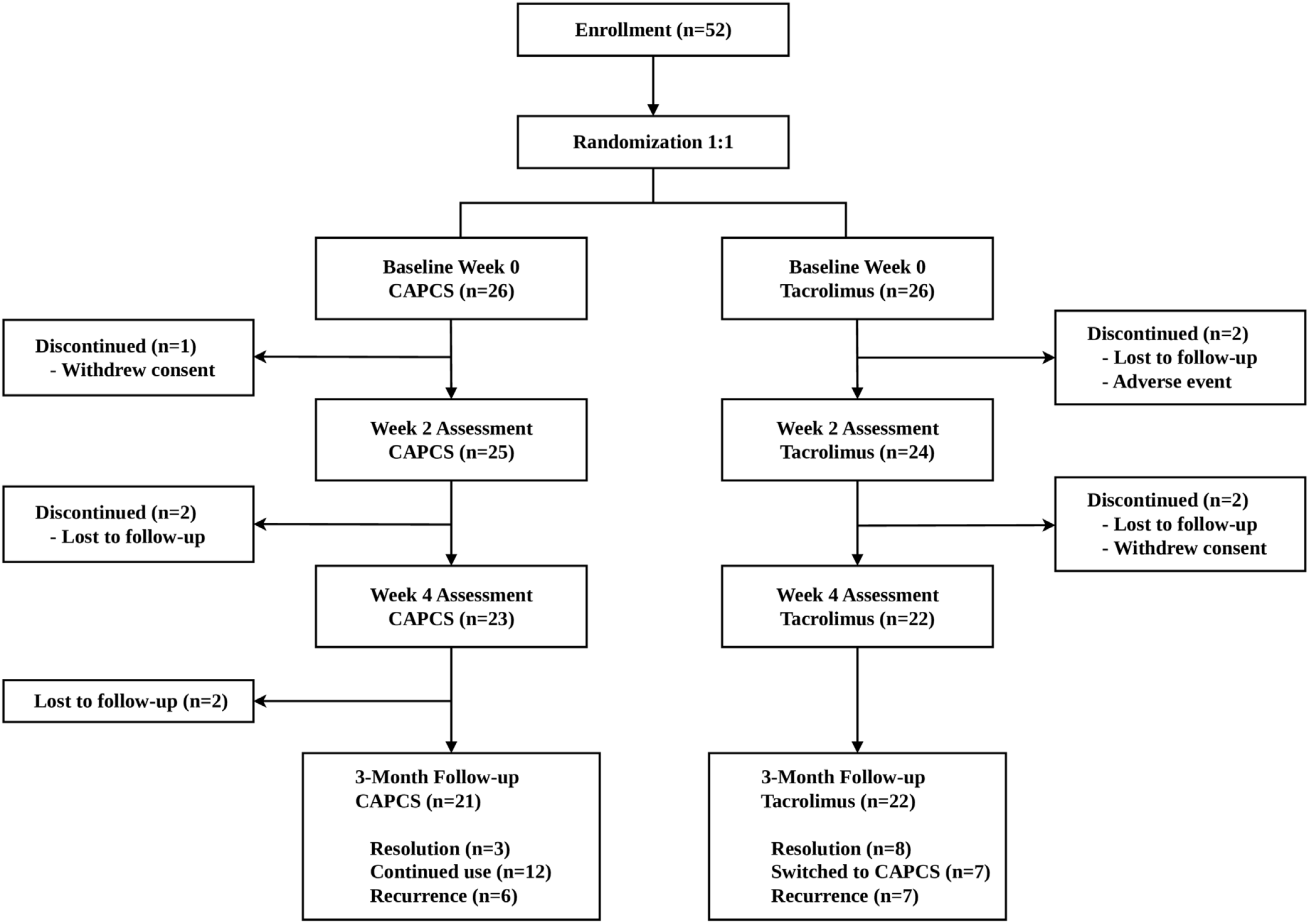
Flowchart and patient status at 3‐month follow‐up. The diagram illustrates the patient disposition throughout the 4‐week trial phase and the 3‐month clinical status.

Patients with cheilitis of other specific causes, oral mucosal diseases, hypersensitivity to study medications, or irreversible forms of cheilitis (such as actinic, granulomatous, glandular, or plasma cell cheilitis) were excluded. Additional exclusion criteria included major systemic diseases, autoimmune or hematologic disorders, acute infections, severe lip infection, pregnancy or breastfeeding, use of systemic steroids within 2 weeks, or use of systemic immunosuppressants within 4 weeks. To minimize treatment interference, topical medications were stopped for at least 1 week before baseline evaluation.

Baseline data included demographics, disease duration, medical and medication history, possible triggers or habits, and clinical features of the lips. Laboratory tests included total serum IgE, complete blood count, and either specific IgE testing or patch testing when indicated.

Patients were assigned study numbers in chronological order and randomized using computer‐generated random number tables (SAS). The CAPCS lip balm and tacrolimus 0.03% ointment were provided in identical containers to maintain blinding, with the experimental group receiving CAPCS lip balm and the control group receiving tacrolimus 0.03% ointment.

Participants received the allocated treatment twice daily for 4 weeks. Before each application, the lips were appropriately cleansed. The use of fluoride toothpaste, lip cosmetics, irritating foods, and excessive UV or temperature exposure was avoided during the study.

### 2.2 Clinical Assessment

Since there was no established evaluation system for assessing chronic cheilitis, we developed a method to evaluate treatment efficacy by observing clinical signs and symptoms. First, based on previous literature and clinical experience [13, 14], we classified clinical manifestations into nine types: dryness, swelling, burning, fissuring, pain, pruritus, scaling, erosion or crusting, and lesion area. Each category was scored from 0 to 3 according to the criteria in Table 1, following *the Clinical Guidelines for Disease Diagnostics and Treatment Evaluation* [15]. Scores were assigned jointly by one doctor and one patient. The score range was from 0 to 27 points, with higher scores indicating greater severity. Clinical evaluations were conducted at baseline and again at Weeks 2 and 4. Treatment responses were then evaluated using the total scores obtained from Table 1. Based on the efficacy index (EI) formula in Table 2, each patient's improvement from baseline was categorized into four levels. Finally, the effective rate (ER) was calculated using the number of recovered cases and the number of cases with a significant effect.

**TABLE 1:** Scoring criteria for chronic cheilitis.

Parameters	0 points	1 point	2 points	3 points
Lesion area (redness)	No lesion	Redness involving < 1/3 of the lip	Redness involving 1/3 to 2/3 of the lip	Redness involving > 2/3 of the lip
Dryness	None	Mild	Moderate	Severe
Swelling	None	Mild	Moderate	Severe
Burning	None	Mild	Moderate	Severe
Fissuring	None	Mild	Moderate	Severe
Pain	None	Mild	Moderate	Severe
Pruritus	None	Mild	Moderate	Severe
Scaling	None	Involvement < 1/2 of lip	Involvement > 1/2 of lip	Extensive/whole lip
Erosion/crusting	None	Involvement < 2 mm in diameter	Involvement > 2 mm but < 1/2 of lip	Extensive involvement of either upper or lower lip

*Note:* Total score ranges from 0 to 27. The severity of dryness, swelling, burning, fissuring, pain, and pruritus was assessed using a subjective 0–3 scale (0 = none, 1 = mild, 2 = moderate, 3 = severe).

**TABLE 2:** Classification of treatment efficacy and calculation methods.

Grade	EI (%)	Clinical interpretation
Recovery	EI ≥ 90%	Near‐complete resolution of lesions
Significant effect	60% ≤ EI < 90%	Marked improvement
Moderate effect	20% ≤ EI < 60%	Partial improvement
No effect	EI < 20%	Minimal or no improvement

*Note:* EI = (pretreatment total score − posttreatment total score)/pretreatment total score × 100%.

ER = (number of recovery cases + number of significant effect cases)/number of cases × 100%.

Abbreviations: EI, efficacy Index; ER, effective rate.

### 2.3 Safety Assessment

Adverse events were monitored throughout the 4‐week study period. Their occurrence, any actions taken, and outcomes were documented. Adverse reactions included systemic reactions, such as headache, dizziness, allergy, and gastrointestinal discomfort, as well as local symptoms including severe pain, burning sensation, and itching. The frequency and percentage of each adverse event were calculated, while the relationship between adverse events and the study treatments was evaluated.

### 2.4 Statistical Analysis

Statistical analyses were performed on patients' baseline characteristics, baseline values for each treatment group, overall efficacy outcomes, individual signs and symptom scores, and safety assessment results. Each categorical variable was described using numbers and percentages. Continuous variables were presented as mean ± standard deviation (SD) for normally distributed data and as median (minimum–maximum) for non‐normally distributed data; however, for the analysis of changes in clinical signs and symptom scores measured on a 0–3 scale, mean ± SD was used instead of the median, as the narrow range of the scale makes the mean a better reflection of changes over time. Normality was assessed using the Shapiro–Wilk test.

Categorical variables were analyzed using the chi‐square test, with exact tests applied when expected cell counts were less than 5: Monte Carlo exact test (10 000 simulations, seed = 2 000 000) for tables larger than 2 × 2, and Fisher's exact test for 2 × 2 tables. Continuous variables were analyzed using the independent samples *t*‐test or the Mann–Whitney *U* test, as appropriate, and the Wilcoxon signed‐rank test was applied for within‐group changes in each symptom and sign. Effect sizes and 95% confidence intervals (CIs) were calculated where applicable. Analyses were based on available data at each time point, without imputation for missing values. Two‐sided *p* < 0.05 were considered statistically significant. All tests were two‐tailed. All analyses were performed using SPSS version 22 (IBM Corp., Armonk, NY, USA).

## 3 Results

### 3.1 Baseline Characteristics

In total, 52 participants were recruited, with ages spanning 13–58 years. The majority were young adult women (63.5%), and their median disease duration was 24 months in the CAPCS group and 27 months in the tacrolimus group. Overall, 23 participants reported a history of atopic disease. No statistically significant differences were observed between the two groups in baseline characteristics, including demographics, disease‐related factors, medical history, and laboratory findings.

### 3.2 Efficacy Analysis

In this study, 26 patients were assigned to the experimental group and 26 to the control group. Subsequently, seven patients were excluded. Of the seven excluded participants, four participants were lost to follow‐up, one participant discontinued participation due to adverse effects, and the remaining two voluntarily withdrew from the study.

At baseline, the mean total score did not differ significantly between the experimental and control groups, with values of 12.6 ± 4.2 and 11.3 ± 3.3, respectively (Table 3, *p* = 0.204). Two weeks after treatment, the mean total score at 2 weeks (6.7 ± 4.0 vs. 4.4 ± 4.3) was significantly lower in the tacrolimus group compared to the CAPCS group (*p* = 0.028), however, by 4 weeks, both groups showed marked improvement from baseline, with ERs of 66.4% ± 29.4% in the experimental group and 79.1% ± 16.7% in the control group. Differences between groups were not statistically significant in efficacy category distribution (*p* = 0.730), ER (*p* = 0.202), and mean total score (*p* = 0.085). Furthermore, in the CAPCS group, the Paired samples *t*‐test indicated a significant reduction in mean total score from baseline to Week 2 (*t*(24) = 5.897, *p* < 0.001) and from baseline to Week 4 (*t*(22) = 8.087, *p* < 0.001). In the tacrolimus group, significant reductions were also observed from baseline to Week 2 (*t*(23) = 7.864, *p* < 0.001) and from baseline to Week 4 (*t*(21) = 12.962, *p* < 0.001). Representative clinical photographs also showed consistent improvement in erythema, scaling, and fissuring during treatment (Figure 2).

**TABLE 3:** Efficacy outcomes over time in the two treatment groups.

Groups	Time	Total Score, mean ± SD	Recovery (≥ 90%), *n* (%)	Significant (60%–89%), *n* (%)	Moderate (20%–59%), *n* (%)	No effect (< 20%), *n* (%)	ER (%), mean ± SD
CAPCS	Baseline (*n* = 26)	12.6 ± 4.2	—	—	—	—	—
	Week 2 (*n* = 25)	6.7 ± 4.0	1 (4.0)	9 (36.0)	11 (44.0)	4 (16.0)	45.2 ± 33.0
	Week 4 (*n* = 23)	4.2 ± 4.0	6 (26.1)	11 (47.8)	5 (21.7)	1 (4.3)	66.4 ± 29.4
Tacrolimus	Baseline (*n* = 26)	11.3 ± 3.3	—	—	—	—	—
	Week 2 (*n* = 24)	4.4 ± 4.3	4 (16.7)	11 (45.8)	7 (29.2)	2 (8.3)	60.7 ± 29.4
	Week 4 (*n* = 22)	2.3 ± 2.0	8 (36.4)	12 (54.5)	2 (9.1)	0 (0.0)	79.1 ± 16.7
*p*	Baseline	0.204^a^	—				—
	Week 2	0.028^b^	0.459^c^				0.089^a^
	Week 4	0.085^b^	0.730^c^				0.202^b^

*Note:* A total of 52 patients were initially enrolled (26 in each group). Due to dropouts, the number of patients available for analysis decreased to 49 at Week 2 (CAPCS 25, Tacrolimus 24) and 45 at Week 4 (CAPCS 23, Tacrolimus 22). The *p* values (0.459 and 0.730) compare the distribution of EI grades (recovery to no effect) between the two treatment groups.

Abbreviations: CAPCS, calcium‐based antimicrobial peptide compounds; EI, efficacy index; ER, effective rate; SD, standard deviation.

^a^ Independent samples *t*‐test.

^b^ Mann–Whitney *U* test.

^c^ Monte Carlo exact test.

**FIGURE 2:**
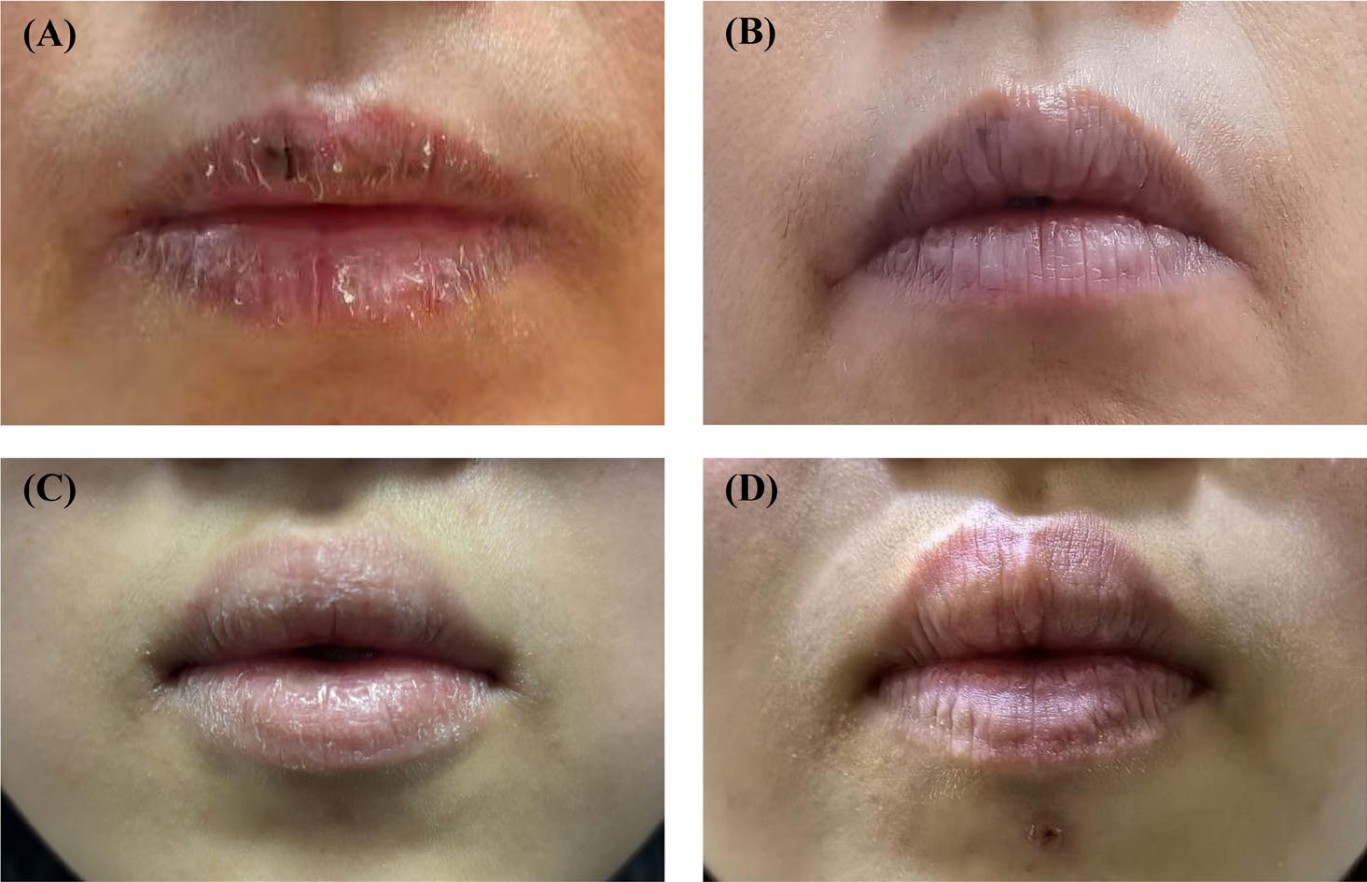
Representative images from two patients, showing their lips before and after 4 weeks of treatment with CAPCS lip balm or 0.03% tacrolimus ointment: (A) Patient 1, CAPCS lip balm, before treatment; (B) Patient 1, after 4 weeks of CAPCS lip balm; (C) Patient 2, 0.03% tacrolimus ointment, before treatment; and (D) Patient 2, after 4 weeks of 0.03% tacrolimus ointment.

We further analyzed changes in clinical signs and symptoms over time. At baseline, lesion area and dryness showed higher severity, with scores of 2.1 ± 0.8 and 2.4 ± 0.7 in the CAPCS group and 2.1 ± 0.8 and 2.2 ± 0.7 in the tacrolimus group (Table 4). Fissuring and scaling presented with moderate severity (CAPCS: 1.7 ± 0.9 and 1.4 ± 0.9; tacrolimus: 1.6 ± 0.9 and 1.8 ± 0.9). Swelling, erosion or crusting, burning, pain, and pruritus showed lower scores, ranging from 0.5 to 1.2 across both groups. Both groups showed significant improvements from baseline to Week 4 in lesion area, dryness, swelling, fissuring, scaling, erosion or crusting, burning, pain, and pruritus (all *p* < 0.05; Figure S1).

**TABLE 4:** Changes in scores of clinical signs and symptoms over time.

Parameters (0–3)	Groups	Baseline, mean ± SD	After 1 month, mean ± SD	*p* (vs. baseline)
Signs				
Lesion area	CAPCS	2.1 ± 0.8	0.8 ± 0.7	< 0.001
	Tacrolimus	2.1 ± 0.8	0.3 ± 0.5	< 0.001
Dryness	CAPCS	2.4 ± 0.7	0.9 ± 0.9	< 0.001
	Tacrolimus	2.2 ± 0.7	1.0 ± 0.7	< 0.001
Swelling	CAPCS	1.1 ± 0.9	0.2 ± 0.5	0.001
	Tacrolimus	0.5 ± 0.8	0.1 ± 0.3	0.035
Fissuring	CAPCS	1.7 ± 0.9	0.4 ± 0.5	< 0.001
	Tacrolimus	1.6 ± 0.9	0.2 ± 0.4	< 0.001
Scaling	CAPCS	1.4 ± 0.9	0.8 ± 0.9	0.017
	Tacrolimus	1.8 ± 0.9	0.3 ± 0.5	< 0.001
Erosion/crusting	CAPCS	0.9 ± 0.7	0.2 ± 0.4	0.002
	Tacrolimus	0.7 ± 0.8	0.1 ± 0.3	0.006
Symptoms				
Burning	CAPCS	1.2 ± 1.0	0.1 ± 0.5	0.001
	Tacrolimus	0.6 ± 0.8	0.1 ± 0.4	0.011
Pain	CAPCS	0.9 ± 0.8	0.3 ± 0.7	0.004
	Tacrolimus	0.8 ± 0.8	0.1 ± 0.2	0.001
Pruritus	CAPCS	1.1 ± 1.3	0.4 ± 0.7	0.006
	Tacrolimus	0.9 ± 1.2	0.2 ± 0.5	0.011

*Note:* Seven participants were excluded prior to the 4‐week follow‐up, resulting in 45 participants completing the 4‐week treatment. At baseline, the CAPCS group included 26 patients, with 23 remaining at Week 4. The tacrolimus group included 26 patients at baseline, with 22 remaining at Week 4. Clinical features were evaluated using a 0–3 scale (0 = none, 1 = mild, 2 = moderate, 3 = severe), and values are presented as mean ± SD. *p* values were calculated using the Wilcoxon signed‐rank test.

Abbreviations: CAPCS, calcium‐based antimicrobial peptide compounds; SD, standard deviation.

### 3.3 Safety Analysis

Eight patients experienced adverse events during the trial period. In the tacrolimus group, 6 of 26 patients (23.1%) experienced adverse reactions, while 2 of 26 patients (7.7%) in the CAPCS group had mild pruritus. The reactions included itching, stabbing pain, and numbness. The adverse events subsided on their own as therapy proceeded, and no further action was required. None of the patients experienced serious adverse events. Although more cases occurred in the tacrolimus group, the difference was not statistically significant (Fisher's exact test, *p* = 0.248).

### 3.4 Three‐Month Follow‐Up

Forty five patients who completed the treatment were followed up at 3 months posttreatment. In the group treated with CAPCS lip balm, 23 patients who completed the 4‐week treatment were followed up. Two patients could not be reached, and three patients reported complete resolution. Twelve patients confirmed that they purchased the CAPCS lip balm and continued to use it, with their lips remaining in good condition due to the continued use. Six patients reported recurrent cheilitis; these patients were not regularly using the CAPCS lip balm at follow‐up.

In the group that used tacrolimus ointment 0.03%, 22 patients were followed up, and 8 patients reported complete resolution. We recommended using CAPCS lip balm for patients who did not achieve complete resolution or who wanted to maintain lip care after the 4 weeks of treatment. Seven patients reported that they purchased the CAPCS lip balm and that applying the balm kept their lips in good condition. Seven patients reported that they still experienced recurrent cheilitis. The follow‐up data are shown in Figure 1.

## 4 Discussion

Chronic cheilitis represents a clinical challenge that warrants greater attention in dermatological practice. Lips are characterized by features of both skin and mucosal tissues, making them particularly prone to inflammation and concomitantly complicating treatment selection [10, 16]. This study is the first controlled trial to evaluate the efficacy and safety of CAPCS lip balm in patients with chronic cheilitis, compared with the established topical treatment, tacrolimus 0.03% ointment. The tacrolimus group showed a faster reduction in mean total score at Week 2 than the CAPCS group, which indicates that tacrolimus elicits a more rapid response. By Week 4, however, the difference between the two treatments was not statistically significant, and both showed marked improvements, with ER values of 66.4% and 79.1%, respectively. Consistently, all nine symptoms and signs showed a clear decrease over time in both groups.

A notable advantage of CAPCS lip balm observed in this study was its favorable tolerability profile. The sole adverse event in the CAPCS group was mild pruritus, which resolved spontaneously without intervention. The favorable tolerability of CAPCS appears to result from its naturally derived ingredients, such as calcium extracted from marine shellfish and AMPs. At the 3‐month follow‐up, the tacrolimus group demonstrated a trend toward higher rates of complete resolution and fewer recurrences compared with CAPCS. However, CAPCS offered an advantage for long‐term maintenance. Patients initially treated with tacrolimus were later switched to CAPCS, and those continuing CAPCS in both groups showed ongoing improvement without safety concerns. Given the long‐term recurrence of chronic cheilitis and the side effects of tacrolimus, these findings support a new two‐step maintenance therapy approach: tacrolimus for rapid effect, followed by CAPCS for safe, long‐term maintenance.

Topical tacrolimus is a calcineurin inhibitor that has been extensively studied and widely used in dermatology since its approval for atopic dermatitis in Japan in 1999. Tacrolimus has demonstrated safety in both cutaneous and mucosal applications, and several reports have documented its successful use in different forms of cheilitis [7, 8, 9, 14]. Despite its efficacy, topical tacrolimus may cause adverse effects that should be considered. Rare but serious concerns include potential systemic absorption and long‐term safety, with the FDA issuing a warning in 2006 about a possible association with lymphoma or skin cancer, although epidemiological studies have not confirmed this link [17, 18]. Other uncommon adverse effects include contact dermatitis, rosacea‐like dermatitis, acne‐like eruptions, and localized flushing occurring after alcohol consumption [19, 20, 21]. The most frequently reported local reactions are burning, pruritus, and numbness at the application site, typically occurring during the initial treatment period. In most cases, these responses decrease with continued use and rarely require discontinuation [18]. Nevertheless, such initial reactions may affect treatment adherence and patient satisfaction in some individuals. In our study, one patient discontinued treatment due to such discomfort. Although serious adverse effects are rare, these considerations highlight the need for alternative treatments with improved safety for long‐term management of chronic cheilitis.

CAPCS represents a novel therapeutic approach that combines natural active calcium derived from marine shellfish with various AMPs. The rationale for using CAPCS in inflammatory skin conditions lies in its multifaceted mechanism of action. In the skin, calcium concentration gradually increases from the lower layers to the upper stratum corneum, forming a gradient that directly regulates keratinocyte migration and differentiation [22, 23]. Calcium ions also play a broader role in maintaining skin barrier stability, promoting wound healing, and modulating local immune signaling [22, 23, 24]. In addition, AMPs are naturally present in the human body and play a role in immune defense [25]. Beyond their antimicrobial effects, they also help modulate inflammatory responses, promote wound healing, and strengthen the skin barrier [25, 26]. Therefore, the combination of calcium and AMPs may act synergistically in addressing multiple aspects of inflammatory skin diseases. Li et al. previously reported that CAPCS gel use in patients with mild to moderate atopic dermatitis led to a reduction in overall disease burden and provided notable relief from pruritus, contributing to better quality of life [12]. Furthermore, the study by Liu et al. demonstrated the anti‐inflammatory activity of CAPCS and showed improvement in contact dermatitis–like skin lesions, which was associated with Th cell response modulation [11]. To date, no serious side effects associated with CAPCS have been reported, which is consistent with its composition of safe, naturally derived ingredients.

To date, studies have also been conducted on non‐pharmacological alternative treatments for various subtypes of cheilitis. While studies on photodynamic therapy (PDT) have primarily focused on actinic cheilitis [27, 28], research has also been conducted on other subtypes [29, 30]. Laser therapy and narrowband ultraviolet B (UVB) therapy have also been reported for exfoliative cheilitis [31, 32]. The use of polarized light therapy for chronic oral mucosal pain has also been reported [33]. However, the evidence supporting these methods is limited, and some reports are based on pilot studies or small‐scale trials. Furthermore, the applications of these treatments were limited. For example, some were used only in patients who did not respond well to existing treatments. While these results suggest that non‐pharmacological alternative treatments have been examined for various forms of cheilitis, the differences among these studies make it difficult to compare them using a single standard. This study focused on chronic cases of mostly reversible cheilitis according to the classification proposed by Lugović‐Mihić et al. Our study was not limited to a single specific subtype. Therefore, direct comparison of CAPCS with previously reported non‐pharmacological alternative treatments remains challenging.

Our findings demonstrated that both treatments significantly improved clinical manifestations after 4 weeks, with CAPCS lip balm showing comparable efficacy to tacrolimus and a favorable tolerability profile. The number of patients withdrawn during treatment in our study was relatively high, primarily due to loss to follow‐up rather than treatment‐related issues. Importantly, since the patterns did not differ significantly between the two groups, the likelihood of meaningful bias in either treatment is low. No significant difference in the incidence of adverse events was observed, and larger studies are needed to further establish the safety of CAPCS compared with tacrolimus. Moreover, as a novel therapeutic agent, CAPCS has limited published clinical data, particularly in the treatment of cheilitis. This study provides initial evidence for its efficacy and safety, but further research with larger sample sizes and longer follow‐up periods is needed to establish its role in clinical practice.

## 5 Conclusion

CAPCS lip balm demonstrated efficacy comparable to 0.03% tacrolimus ointment after 4 weeks of treatment and showed excellent tolerability. We confirmed that CAPCS lip balm can serve as a reliable alternative treatment option for patients experiencing discomfort from tacrolimus‐related side effects or requiring long‐term management. Additionally, a sequential treatment approach, in which tacrolimus ointment is used for rapid initial improvement followed by maintenance with CAPCS lip balm, suggests that it can help maintain patients' lip health while minimizing the side effects associated with long‐term calcineurin inhibitor use.

## Author Contributions

H.J. collected the data, performed the statistical analysis, and wrote the manuscript. T.C. conceived and designed the study and contributed to data collection. S.C., J.X., S.G., and A.D.S. contributed to data collection. All authors reviewed and approved the final manuscript.

## Funding

This work was supported by HEFEI Shell Party Innovation Technology Co. Ltd.

## Ethics Statement

This study was approved by the Institutional Review Board of The First Affiliated Hospital of Chongqing Medical University and complied with the Declaration of Helsinki and the laws and regulations of China.

## Consent

Written informed consent was obtained from all participants.

## Conflicts of Interest

The authors declare no conflicts of interest. Hefei Shell Party Innovations Co. Ltd. provided products and support for this study but did not participate in data collection, analysis of results, or manuscript preparation.

## Supporting information


**Figure S1:** Changes in clinical sign and symptom scores from baseline to Week 4 in the CAPCS and tacrolimus groups. Scores for lesion area, dryness, swelling, fissuring, scaling, erosion/crusting, burning, pain, and pruritus were assessed at baseline and Week 4. Both treatment groups showed significant reductions in all clinical sign and symptom scores from baseline to Week 4 (all *p* < 0.05).

## Data Availability

The data that support the findings of this study are available on request from the corresponding author. The data are not publicly available due to privacy or ethical restrictions.
